# Development and Feasibility of a Novel mHealth Resource for Food Insecurity: Mixed Methods Cohort Study

**DOI:** 10.2196/65852

**Published:** 2025-08-26

**Authors:** Cristin Q Fritz, Meng Xu, Justine Stassun, Susana Martinez, Laura M Gottlieb, William J Heerman, Derek J Williams, Lindsay S Mayberry

**Affiliations:** 1 Department of Pediatrics, Vanderbilt University Medical Center, Office #118, 2141 Blakemore Avenue, Nashville, TN, 37212, United States, 1 615-936-9263, 1 615-875-4623; 2 Department of Biostatistics, Vanderbilt University Medical Center, Nashville, TN, United States; 3 Vanderbilt Institute for Clinical and Translational Research, Vanderbilt University Medical Center, Nashville, TN, United States; 4 Department of Family and Community Medicine, University of California, San Francisco, San Francisco, CA, United States; 5 Department of Internal Medicine and Public Health, Vanderbilt University Medical Center, Nashville, TN, United States

**Keywords:** food insecurity, mobile health, mHealth, pediatric hospitalization, food resources, health related social needs

## Abstract

**Background:**

Pediatric clinical practice guidelines recommend identifying and addressing food insecurity (FI) as part of routine care. However, methods for health systems to connect families experiencing FI to community food resources are lacking. Confidential SMS text messaging can increase equity in resource delivery, is user-friendly, is aligned with caregiver preferences, and is feasible for health systems to implement. Despite the promise of this approach, SMS text messaging has not been widely tested in pediatric settings.

**Objective:**

This paper details (1) the process of developing a novel, mobile health intervention to help families access local food resources and (2) results on reach, engagement, usability, and acceptability of the intervention following a 1-year pilot.

**Methods:**

We designed and evaluated an automated SMS text messaging system that delivers geographically tailored food resource information to families with FI after hospital discharge at a single US children’s hospital. English- and Spanish-speaking caregivers of hospitalized children with a positive FI screen documented during clinical care were included. Caregivers received a food resource text message 1 and 4 days postdischarge. In addition, 2 subsequent text messages asked about reach and engagement. We used system-reported (primary) and caregiver-reported (secondary) measures of reach and engagement and caregiver-reported resource connection as a preliminary measure of effectiveness. We assessed usability (Simplified System Usability Scale [SUS]; >75 indicates good usability), acceptability, and caregiver preferences for resource provision through semistructured interviews among a subset of caregivers (20 English-speaking and 11 Spanish-speaking caregivers).

**Results:**

Of 194 patients with a positive FI screen during the study period, 187 (96%) spoke English or Spanish and were included in the cohort. Primary, system-reported measures indicated that the food resource message successfully reached 175 (94%) participants; of these, 102 (58%) engaged with the text messages in some way, with 65 (37%) clicking the link and 92 (53%) responding to a text message. Among the subset of text message respondents (n=92), 88 (96%) reported receiving the resource message, 83 (90%) read the message, and 42 (46%) used the information to search for food resources. Among the subset of interviewed caregivers (n=31), the median SUS score was 86.1 (IQR 66.7‐91.7); 97% (30/31) of caregivers felt the intervention was acceptable. Caregivers preferred receiving food resource information via text message rather than paper handouts because it felt more accessible.

**Conclusions:**

Providing automated, geographically tailored food resource information via text message to families with FI after hospital discharge was feasible, the information was usable, and the delivery mode was acceptable to families, with SMS text messaging preferred over paper handouts. SMS text messaging offers a promising low-intensity approach to social resource provision for health systems. Future research should assess effectiveness and strategies to increase uptake in clinical care contexts.

## Introduction

Childhood food insecurity (FI)—the lack of consistent access to enough food to lead an active and healthy lifestyle [1]—is associated with negative health outcomes such as poorer overall health; increased likelihood of having diabetes and hypertension as adults; and higher rates of anxiety, depression, and behavioral dysregulation [2-5]. In addition, FI disproportionately impacts families who are low-income, immigrants, and non-White, as well as single-parent households, thereby contributing to disparities in health outcomes [1,6]. Hospitalized children and their families are particularly vulnerable to FI, given the often unexpected financial stress resulting from hospitalization, including from both hospital costs and potential for lost caregiver wages [7-9].

Connecting families with supplemental food resources, such as federal nutrition programs, can improve family food security and child health outcomes [10,11]. However, little is known about effective methods for facilitating those connections to relevant food resources [12-16]. Though newer, web-based community resource referrals platforms are increasingly available across the United States, in children’s hospitals, the most commonly reported strategies used to connect families with community resources include use of a standardized paper resource handout, as well as consultation with a social worker or other in-person resource navigator [17-19]. Barriers to the effectiveness of these approaches have been described in the pediatric literature, including reliance on (1) generic paper handouts, which are often not tailored to a patient’s location, outdated or incomplete, and easily misplaced; and (2) patient discussions with a care team member, which may dissuade families from seeking help due to concerns about stigma and discrimination and can require significant hospital resources [13].

SMS text messaging interventions may offer one way to overcome these barriers to sharing food resource information. Though disparities exist in smartphone ownership, patient portal access, and mobile application use [20-22], basic cell phones are nearly ubiquitous among US adults [21], and SMS text messaging is common among cell phone owners of all income levels and racial or ethnic backgrounds [23-26]. Families surveyed in acute care settings have indicated a preference for receiving customized resource information via text message [18,27]. Moreover, a range of health content delivered via SMS text messaging has been shown both to reach and improve outcomes among underserved or traditionally harder-to-reach populations [24-26]. Thus, providing food resource information via text message may enable health systems to improve both equity and sustainability of food security interventions [28]. However, text message-based approaches to providing resource information have not been widely utilized or rigorously evaluated.

To fill this gap and overcome previously described barriers to information-based, food security–related interventions, we developed Text Connect, a mobile health (mHealth) intervention that sends an automated text message with ZIP code–specific food resource information at discharge to families with FI identified during hospital admission. This study aimed to evaluate the reach, engagement, usability, and acceptability of this novel, automated text message intervention among hospitalized children and their families.

## Methods

### Study Design and Inclusion Criteria

We conducted a mixed methods cohort study at Monroe Carell Jr. Children’s Hospital at Vanderbilt, a single, freestanding, tertiary care children’s hospital from February 2023 to February 2024. English- and Spanish-speaking caregivers of children 18 years or younger admitted to a pediatric hospital medicine team with a positive inpatient FI screen on the 2-question Hunger Vital Sign screener (answer of “Sometimes“ or “Often” to either question) were eligible for study inclusion. Only the first encounter was included for patients with multiple admissions during the study period. We aimed to include a cohort that was representative of the patients with positive FI screens admitted to our hospital, thereby supporting our goal to understand the feasibility of our automated, pragmatic approach.

### Study Context: Hospital Population and Food Insecurity Screening Process

This study took place at an academic tertiary care pediatric hospital located in the Southeast region of the United States. The hospital’s patient population included children from urban, suburban, and rural areas across 9 states. In 2023, over half (54%) of admitted patients were insured by Medicaid. English (87.6%), Spanish (9.1%), and Arabic (1.4%) were the 3 most common preferred languages. The prevalence of FI in the state in 2023 was 11.7% [29].

Inpatient FI screening was implemented as part of routine clinical care at the hospital in 2021. Screening was iteratively expanded and adapted using Quality Improvement methodology to reach the goal of sustainably screening 80% of eligible families. The full details of the hospital’s screening process and associated outcomes are detailed in a previous publication [30]. At the time of this study, families admitted to one unit of the hospital were routinely screened for FI with the validated 2-question Hunger Vital sign [31,32]. Screening occurred at the time of nursing admission intake; participant responses were entered into the electronic health record (EHR). Caregiver contact information was also reviewed as part of the nursing admission process. All families with documented FI were provided information at discharge in their preferred language that included a food resource handout containing general information on federal support programs (eg, Supplemental Nutrition Assistance Program [SNAP]) and links to ZIP code–based searches for emergency community food resources. The discharging nurse was responsible for providing and reviewing this information at the time of hospital discharge [30]. The Text Connect intervention was added to this existing workflow for the duration of the study. All other aspects of the workflow were unchanged.

### Text Connect Development

#### Message Content

We developed the initial text message content using information frequently provided to families by the hospital’s social work team. Content was refined through a design studio led by Vanderbilt’s Community Engaged Research Core that uses direct feedback from community members (“experts”) who share similar demographic characteristics with a researcher’s desired sample to identify and address concerns and provide advice [33]. In this instance, the community experts included caregivers of children with FI identified during a recent hospital admission. This group confirmed that caregivers preferred SMS text messaging to other communications as a method to receive postdischarge resources, reviewed and approved messaging content and wording, and provided recommendations for timing of message delivery. Draft content was also reviewed by Vanderbilt’s Effective Health Communication Core and updated to ensure alignment with best practices for effective communication and readability metrics.

**Figure 1.**
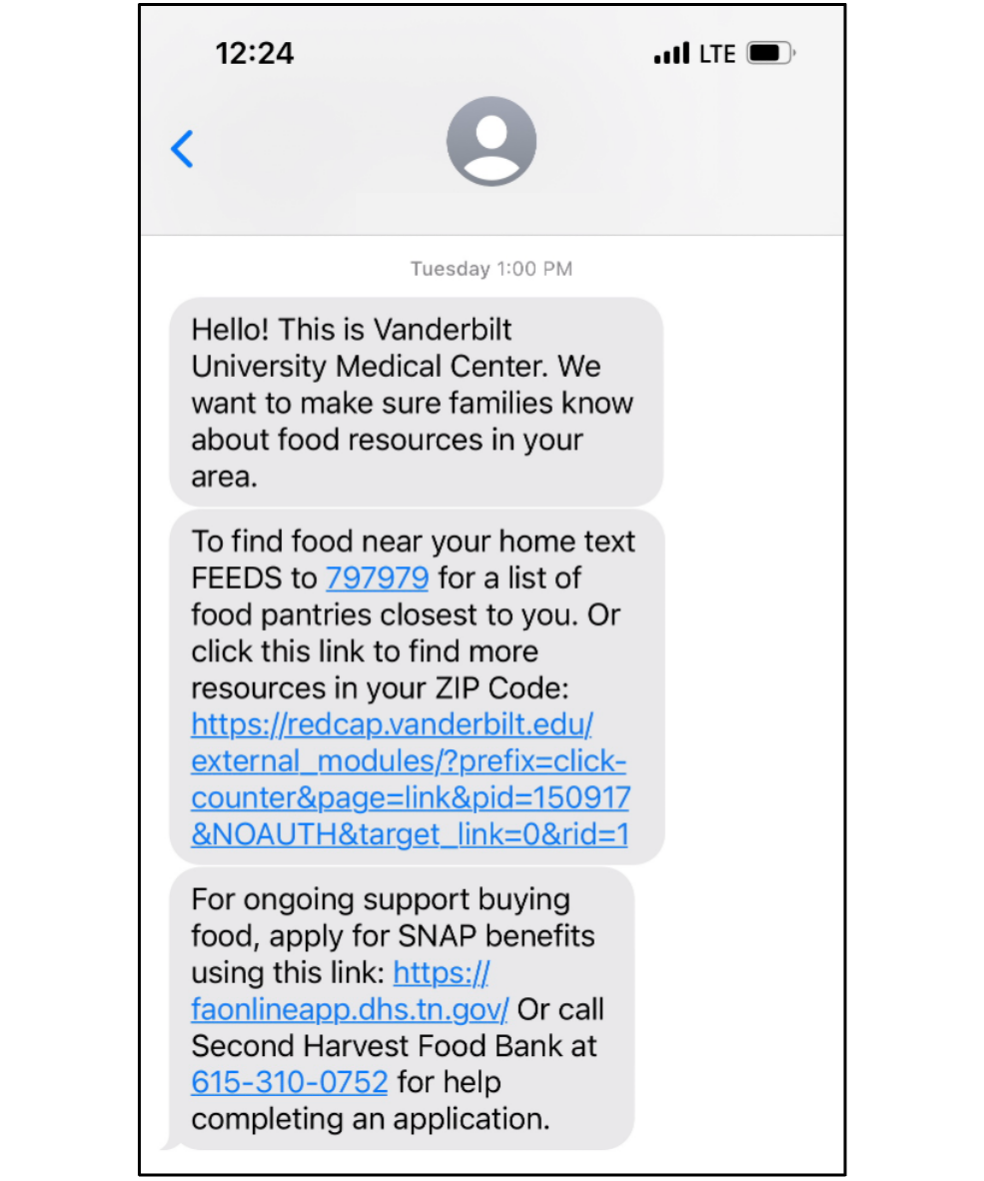
Text Connect message prototype.

The final Text Connect message included three primary components: (1) a link to a FindHelp [34] food resource search (online social resource database available in over 100 languages) tailored to the recipient’s home ZIP code; (2) instructions for receiving ZIP code–specific emergency food resource information through text message utilizing an existing system managed by a regional food bank; and (3) information regarding applying for the SNAP online or with assistance from a SNAP outreach specialist at the food bank (Figure 1). This combined approach was designed to ensure families without internet access or adequate cell phone data (required to search through the FindHelp link) could nonetheless receive location-specific resources. All text message content was at or below a 6th grade reading level and available in English and Spanish languages. A reminder message with the same content was sent 3 days after the initial one to provide another opportunity for families to receive and engage with the information. Families could engage with the information in the resource message by (1) clicking on the included FindHelp link, (2) clicking on the SNAP link, (3) texting the food bank system, or (4) calling for assistance with a SNAP application.

#### Message Automation

**Figure 2.**
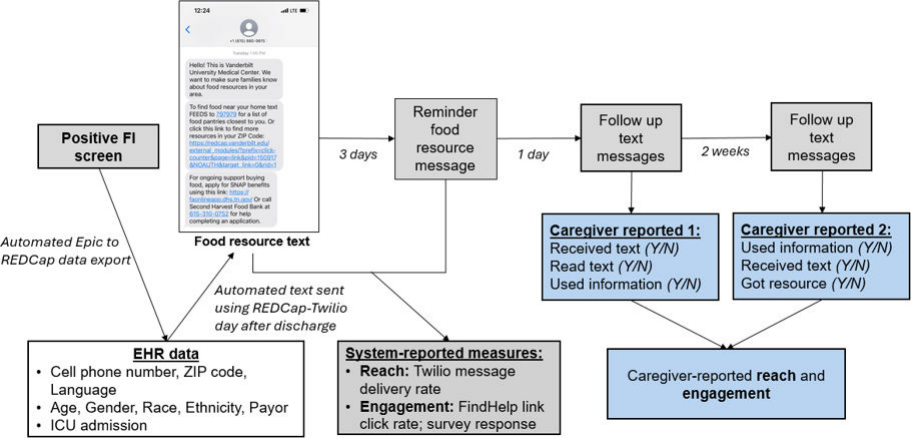
Text Connect intervention delivery and data collection. EHR: electronic health record; FI: food insecurity; ICU: intensive care unit; REDCap: Research Electronic Data Capture.

Three components were required to send automated text messages after in-hospital FI screening occurred. Each component was developed with input from a multidisciplinary team. First, an EHR report was created to capture all patients discharged from the hospital in the prior 24 hours with a documented positive FI screening result. The report included demographic data needed to send the text message (cell phone number, preferred language, ZIP code) and to describe the included cohort (patient age, gender, race, payor, intensive care unit admission, length of stay, and FI screening question responses). Using an automated module built for this study, the reports were exported nightly to a secure REDCap (Research Electronic Data Capture; Vanderbilt University) database [35] (online data management platform) with a new record created for each newly eligible patient. Finally, preferred language and ZIP code were used to automatically generate a ZIP code–specific FindHelp search URL that was included in the custom text message sent to each participant’s cell phone using the REDCap to Twilio [36] (third-party messaging service) interface the day after hospital discharge (Figure 2). We also included a REDCap click-tracking module associated with the FindHelp link that recorded a count any time the link was clicked. Before study initiation, we completed end-to-end Beta testing of this process among study team members and addressed all identified issues with message delivery.

#### System Monitoring

The study team developed a process for weekly monitoring of the REDCap data to ensure all technology components (EHR report, REDCap export, and Twilio message) were functioning as expected. The project team was promptly notified about any concerns, such as unanticipated missing data, component failures, or reporting errors, to identify and address the source of system breakdown.

### Study Procedures

#### Text Connect Delivery and Data Collection

Participants were identified for study inclusion through an automated daily EHR report (described in the *Message Automation* section). Participants received a maximum of 4 automated text message encounters in their preferred language. The intervention consisted of the initial Text Connect message and a reminder message containing identical information 3 days later [37]. Because we intentionally included food resource information that could be accessed without internet or cellular data, we were unable to fully capture engagement with all message components among the full cohort. Thus, two different text messages, sent 1 day and 2 weeks after the reminder Text Connect message, gathered caregiver-reported data on reach, engagement, and new resource connections (Figure 2 and Multimedia Appendix 1). Manual EHR review was also performed postdischarge to collect descriptive data missing from the automated REDCap data extractions (defined in the *Message Automation* section).

#### Semistructured Interviews

From the included cohort, we recruited 31 English- and Spanish-speaking caregivers to participate in semistructured, audiotaped interviews. We aimed to recruit both caregivers who did and did not engage with text messages. The interview guide (Multimedia Appendix 2) was developed using the Technology Acceptance Model for Resource-Limited Settings (TAM-RLS) framework [38,39] through consultation with content area and methodology experts and translated into Spanish by a certified interpreter. Interview content included structured survey questions with follow-up probes designed to assess the usability and acceptability of the intervention, understand how participants engaged with the provided information, and solicit input on how to improve the relevance of information in the text message. We also asked open-ended questions to understand caregivers’ general perspectives on receiving resources through text message. The interview guide was tested among caregivers on an existing FI advisory council (led by author CQF) using concurrent think-aloud technique [40] before use with study participants. Interviews were conducted by a trained research assistant unknown to participants over the phone in the participant’s preferred language (English or Spanish). Interviews were also transcribed and translated into English, when applicable.

### Measures

To evaluate the feasibility of the Text Connect process and acceptability of the intervention, we used (1) primary measures of system-reported reach and engagement among the cohort of all caregivers included in the study (henceforth “included cohort”); and (2) secondary measures of caregiver-reported reach and engagement among a subset of caregivers who responded to text messages (henceforth “text message respondents”). We also assessed usability and acceptability through semistructured interviews with a subset of English- and Spanish-speaking caregivers.

We defined the primary, system-reported measure of the intervention’s reach as the proportion of the included cohort who received the Text Connect message based on Twilio message delivery data. Twilio reports the status of each message (ie, sent, delivered, undeliverable) and generates error notifications for any undeliverable message (eg, number is not in service, landline that cannot receive text messages, etc). Among text message respondents, a secondary, caregiver-reported measure of intervention reach was defined by caregiver report of receiving the Text Connect messages.

We defined the primary, system-reported measure of intervention engagement as the proportion of included caregivers that either clicked the FindHelp link or responded to at least one text message. This combined measure was selected to be inclusive of caregivers who engaged with the Text Connect system by responding via text message but who may have been unable or chose not to use the embedded FindHelp link. Among text message respondents, we defined secondary measures of caregiver-reported engagement as (1) the proportion of caregivers who reported reading the text message; and (2) the proportion of caregivers who used the information to search for food resources.

We assessed resource connection as a preliminary measure of effectiveness. Among text message respondents who reported using the included information to look for a resource, we defined resource connection as the proportion of caregivers that reported getting a new food resource since their child’s hospitalization.

Interview participants completed the validated, 9-question Simplified System Usability Scale (SUS) to quantify intervention usability. The SUS is scaled from 0 to 100, with scores >75 indicating good usability [41]. Open-ended follow-up questions were asked to characterize identified usability issues. Acceptability was assessed among interview participants based on caregiver agreement with the statement, “The text message is an acceptable way to get food resource information.”

### Analysis

We used descriptive statistics to summarize primary and secondary measures as well as clinical and sociodemographic characteristics of the included cohort, those receiving Text Connect based on the system-reported measure, and those responding to follow-up messages or clicking the FindHelp link. We compared demographic variables between text message respondents and nonresponders using the Wilcoxon rank sum test for continuous variables and the Pearson chi-square test for categorical variables. Usability scores were scaled to 100 for comparison against established benchmarks and summarized for the interviewed cohort. For qualitative analyses, we used rapid thematic analysis to (1) explore caregiver preferences for receiving food resource information through text message compared to typical methods of paper handouts and meeting with a person in the hospital (ie, a resource navigator); and (2) identify usability issues and barriers to engagement with the intervention. Primary themes were informed by quantitative data and inductively assigned by PI (CQF) and reviewed by LSM and DW. Themes were compared between English- and Spanish-speaking caregivers. We used triangulation between quantitative outcomes and themes that emerged between English and Spanish caregivers to ensure the trustworthiness of qualitative analyses. Quantitative analyses were conducted using R statistical software.

### Ethical Considerations

This study posed no greater than minimal risk to participants and was deemed exempt from review by the Vanderbilt institutional review board (#222183) based on analysis of deidentified data. Informed consent was waived due to the low-risk nature of the study and approval under “Exempt” status by the institutional review board. Privacy and confidentiality of participants were maintained through data storage on password-protected servers and analysis of deidentified data. All interview participants received a US $30 gift card as compensation for their time.

## Results

### Included Cohort

**Figure 3.**
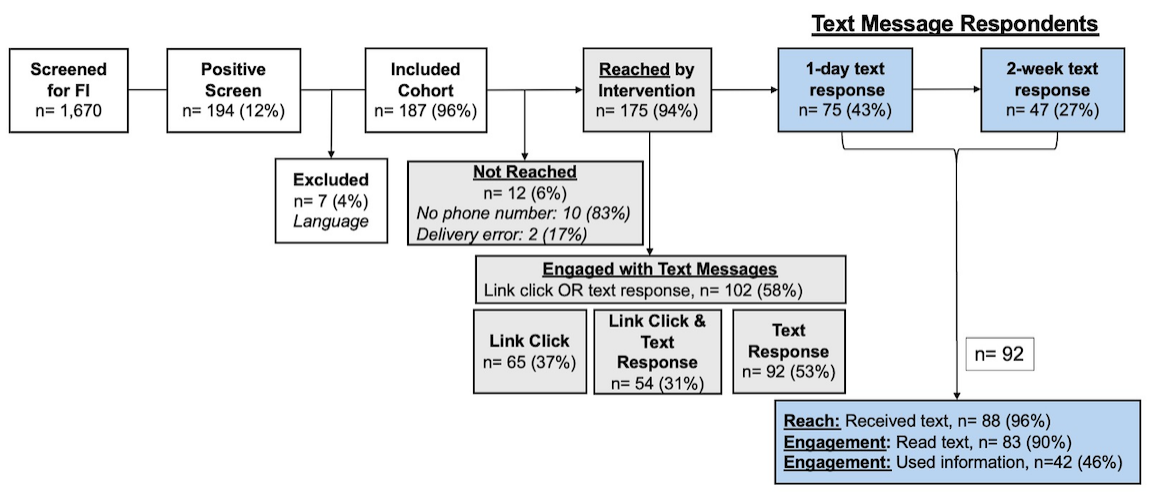
Study inclusion and outcomes. FI: food insecurity.

During the study period, 1670 patients on pediatric hospital medicine teams were screened for FI. Of those screened, 194 (194/1670, 12%) had a positive screen for FI, and 187 (187/194, 96%) of these preferred English or Spanish and were included in the study cohort (Figure 3). Most participants reported non-White race (107/187, 60%), were publicly insured (165/187, 90%), preferred English (133/187, 71%), and lived in a nonrural ZIP code (137/187, 75%) (Table 1).

**Table 1.** Patient demographic and clinical characteristics.

	Included cohort (n=187)	Reached by intervention (n=175)	Engaged with text messages^a^	
			Text message respondents (n=92)	Clicked FindHelp link (n=65)
Age (years), median (IQR)	1.3 (0.3‐5.0)	1.2 (0.3‐5.0)	1.4 (0.2‐6.0)	1.6 (0.3‐6.0)
Female, n (%)	83 (44.0)	82 (46.9)	46 (50.0)	32 (49.2)
Race^b^, n (%)				
African American	31 (17.0)	31 (18.6)	18 (20.5)	10 (16.4)
Hispanic/Latino	56 (31.3)	50 (29.2)	35 (39.8)	25 (41.0)
White	72 (40.2)	66 (39.5)	27 (30.7)	22 (36.1)
Multiple	12 (6.7)	12 (7.2)	6 (6.8)	2 (3.3)
Other	8 (4.5)	8 (4.8)	2 (2.3)	2 (3.3)
Language, n (%)				
Spanish	54 (28.9)	49 (28.0)	35 (38.0)	25 (38.5)
English	133 (71.1)	126 (72.0)	57 (62.0)	40 (61.5)
Rurality, n (%)				
Urban	137 (74.5)	130 (75.6)	71 (77.2)	52 (81.2)
Rural	47 (25.5)	42 (24.4)	21 (22.8)	12 (18.8)
Primary payor, n (%)				
Commercial	9 (4.9)	9 (5.2)	4 (4.4)	1 (1.6)
Public	165 (89.7)	153 (89.0)	83 (91.2)	60 (93.4)
None	5 (2.7)	5 (2.9)	3 (3.3)	2 (3.1)
Tricare	5 (2.7)	5 (2.9)	1 (1.1)	1 (1.6)
Intensive care unit admission, n (%)				
Yes	39 (20.9)	37 (21.1)	19 (20.7)	15 (23.1)
Social risk, n (%)				
Sometimes experience food insecurity^c^	168 (89.8)	156 (89.1)	82 (89.1)	59 (90.8)
Often experience food insecurity^c^	19 (10.2)	19 (10.9)	10 (10.9)	6 (9.2)

a Groups are not mutually exclusive, so participants may be included in more than one engagement category.

b As documented in electronic health record.

c In response to Hunger Vital Sign screening questions.

### Text Connect System Operation

Three types of system errors were identified and addressed during the study: (1) failure to import data for certain demographic and clinical variables into the report (10 participants); (2) failure to automatically import data into the REDCap system due to a communication breakdown between the EHR and REDCap following an EHR system update (9 participants); and (3) failure to trigger the initial text due to a REDCap connection error (15 participants). After these system errors were identified during regular quality checks and resolved, the text message series was manually triggered for all affected participants. The affected 24 participants with a phone number in REDCap were subsequently able to participate in other study activities.

### Primary Measures: System-Reported Reach and Engagement

Among the included cohort (n=187), 175 (94%) were reached by the intervention. Twelve (6%) participants were not reached due to (1) phone number did not import into REDCap (n=10, 83%); or (2) an undeliverable message (n=2, 17%) (Figure 3). The reached cohort had similar distributions of demographic and clinical characteristics as the full cohort (Table 1). System-collected measures indicated that 102 (102/175, 58%) of the reached participants engaged with the text message exchange in some way, with 65 (65/175, 37%) engaging by clicking the FindHelp link and 94 (94/175, 53%) responding to at least one message. 54 (54/175, 31%) participants did both. (Figure 3).

### Secondary Measures: Caregiver-Reported Reach and Engagement

Ninety-two (92/175, 53%) of the reached caregivers responded to at least one text message. There was a higher proportion of Hispanic caregivers (40% [35/92] vs 23% [21/95], *P*=.03) and caregivers who preferred Spanish (38% [35/92] vs 20% [19/95], *P*=.01) among text message respondents compared to nonrespondents. Eighty-eight (88/92, 96%) of text message respondents reported receiving the text message with food resources, 83 (83/92, 90%) reported reading the text message, and 42 (42/92, 46%) reported using the information to look for a new resource (Figure 3).

### Resource Connection

Questions were asked in a sequential, nested fashion such that only caregivers who reported using the included information were asked about connection to a new resource. Among the 42 text message respondents asked about connection to a food resource, 7 of the 11 participants (64%) with a response to the relevant question reported getting a new food resource.

### Usability and Acceptability

We interviewed 20 caregivers who preferred English and 11 who preferred Spanish. Most caregivers were female (28/31, 90%), the patient’s mother (28/31, 90%), and non-White (18/31, 60%); 13/31 (42%) reported Hispanic ethnicity. The median caregiver age was 29.5 years (23.3‐38.0) (Table 2).

**Table 2.** Demographic characteristics of interview participants (n=31).

	Value
Age (years), median (IQR)	29.5 (23.3‐38.0)
Female, n (%)	28 (90.3)
Relationship to Patient, n (%)	
Mother	28 (90.3)
Father	3 (9.7)
Race, n (%)	
African American	7 (22.6)
White	13 (41.9)
American Indian	1 (3.2)
Other	10 (32.3)
Ethnicity, n (%)	
Hispanic/Latino	13 (41.9)
Non-Hispanic	18 (58.1)
Language, n (%)	
Spanish	11 (35.5)
English	20 (64.5)

Among interview participants, the median SUS score was 86.1 (66.7‐91.7), indicating excellent usability. In looking at individual items assessed within the SUS, 81% (25/31) felt confident using the text message to look for food resources, and 94% (29/31) would use the information in the text message again. In total, 97% of participants agreed or strongly agreed that SMS text messaging is an acceptable way to receive food resource information. The lowest usability item was whether the text message was “easy to use” (24/30, 80% of respondents agreed or strongly agreed). Issues with usability and acceptability of the intervention included (1) technical issues such as the text or website being in the wrong language or website being difficult to navigate; (2) preference to discuss available resources with a person; and (3) distrust in the information in the text message (eg, wary of scams).

Nearly all caregivers reported a preference for receiving information through text message compared to a paper handout. Reasons included that (1) handouts are easily lost among many papers received at discharge; (2) text messages can be searched directly on a personal phone and are accessible at any time; and (3) text messages felt more personalized. Preferences were mixed when asked about resource connections provided by a resource navigator. Perceived benefits of a navigator included feeling like participants would receive more personalized information and real-time assistance with questions; perceived benefits of a text message included being more private and more convenient when juggling caregiving responsibilities. Illustrative quotations for these themes are included in Tables3  4. Each row in the table represents responses from an individual caregiver.

**Table 3.** Benefits of text message versus getting information on paper.

Theme	Quote (language spoken, intervention engagement)
Paper easily lost, thrown away/destroyed, or not read	“Especially coming out of the hospital...there’s usually a good bit of paperwork, so it could easily just get mixed up in there. And then, by the time you realize it was mixed in with the other ones that got thrown away, it’s too late by then to try to get it back.” (English speaking, engaged with intervention) “I know, personally, all the discharge papers I just put up, instead of reading thoroughly, other than her medical information.” (English speaking, not did engage with intervention) “I received [the papers], and I have them here in the car but I didn’t read them.” (Spanish speaking, did not engage with intervention)
Text always accessible	“And sometimes I’ll misplace paperwork, like, if I do actually write something down. So I guess because you have all your information there, you can kind of refer back to that. That’s nice. having it written down for you, having it like that link, right there, that you can just go back to that text and—Yeah, that would be an advantage.” (English speaking, engaged with intervention) “I know for me, like as soon as I got home, because there wasn’t no document for his hospital papers, I kind of just had it on the table, and my oldest played with it. So, then it was like, dang, that paper is ruined, and I don’t know-- I didn’t write down any of the numbers or anything. But having the text message, you can always just go back to your messages and be like, oh yeah, this was the link, this was this, this, this. And having it in the text message, you can share it to other people that’s in need and stuff like that.” (English speaking, engaged with intervention) “If anybody is like me, papers get lost. They will end up under my car seats. They’ll end up in a stack, a pile somewhere, get lost. And then people don’t get that information, versus just about everybody has their phone...You’ll always have that information on you, if you’ve got your phone, versus, you can’t just carry around a piece of paper for no reason.” (English speaking, engaged with intervention)
Text more personalized	“The text message is better. I perceive that it’s more personal through a text since it shows more interested in wanting to help out.” (Spanish speaking, engaged with intervention) “If there was a paper handout, as a mother, it’s easier to lose. I like the text messages because you can put in your zip code. That’s the big thing, because a lot of places just give you info on paper that’s in their area.” (English speaking, engaged with intervention)

**Table 4.** Benefits of text message versus meeting with in-person navigator.

Theme	Quote (language spoken, intervention engagement)
Text more convenient when juggling caregiving responsibilities	“Personally, it’s nice to have someone face-to-face to talk to about it in person, but I still think the text message is more convenient, especially in my case. My husband’s in the army. I’m a stay-at-home mom with our ten-month-old and my daughter, who’s normally in school, but she’s out for the summer, so I stay home with them.” (English speaking, engaged with intervention) “The text message was the best strategy, in my personal opinion, because I have five children at home, so it helps me the most.” (English speaking, did not engage with intervention)
Text offers privacy	“Well, I guess, [text is] an easier way to get the information because, like, when you talk to somebody, people might not reach out as much because talking to somebody is a little more personal, you know? And maybe, I guess them thinking—because it sucks. It’s kind of embarrassing. Nobody wants to be that parent or that adult that has crap going on...” (English speaking, engaged with intervention)
Navigator provides more personalized information; texts always accessible	“You will have answers to your questions right then and there. And it’s convenient for you because they’re right there, and then they can explain more of what this company does, this company, this company, this company….I still feel like they should receive a text message because, like I said, my two-year-old tore up the paper. So, the person coming in there, talking to you, taking notes, and stuff like that. What if the notes get messed up, and then you’re like, oh, I still don’t remember the sites. The text message would still come in handy.” (English speaking, engaged with intervention)
Navigator provides more personalized information	“It would be, I hope, a lot more personal. Because for me, I live two hours away from [the hospital], so I was down there with my baby and didn’t know anything. Speaking to people about it, that’s more personal.” (English speaking, did not engage with intervention) “Yes, [a community navigator] would have helped me a lot because I am a single mother of two children, since I don’t have a job.” (Spanish speaking, engaged with intervention) “I feel like meeting with someone in person would be a great resource for a lot of families… meeting in person, they will actually be able to walk you through. And if you have questions, they are answered right away. They can help you understand. And there’s more of a connection with the flow of information versus, you’re reading a text message, almost as good as Googling something because you’re still sitting there, like, Okay, well, I don’t know what that means, how do I still find this?” (English speaking, engaged with intervention) “It would be nice if someone would been able to tell you about the program in person and let them know that they are going to send you a text message to keep an eye on it.” (Spanish speaking, did not engage with intervention)

## Discussion

### Principal Results

Our team developed and piloted Text Connect, a novel, automated mHealth intervention to provide ZIP code–specific food resource information to English- and Spanish-speaking families with FI identified during a hospital admission. The Text Connect intervention reached 94% of families with FI, with over half of recipients engaging with the text messages despite no prior communication from the hospital that the text would be sent to them. Caregiver-reported usability and acceptability of the intervention were high, and 64% of text message respondents reported getting a new food resource due to information contained in the text message. Interview participants preferred Text Connect over paper handouts due to the message accessibility and tailoring.

### Comparisons With Previous Work

These results are especially relevant in the context of recent attention to social and medical care integration, where hospital systems across the country are actively implementing social risk screening in response to federal mandates from the Centers for Medicare and Medicaid Services and The Joint Commission [42,43]. Though addressing disclosed social risk information is a key component of many national recommendations, evidence to guide best practices for responding to positive FI screens is lacking [44]. As a result, existing approaches are heterogeneous, many have not been evaluated, and many are person-time intensive or rely on a static, standardized paper handout that is not tailored to patient needs or location [19]. Online referral platforms are increasingly popular and may overcome these limitations, but they also can require substantial investment from the health system and community partners for purchase, implementation, referral, and outcome reporting.

Our study demonstrates that an automated, EHR-generated text message with location-specific resource information offers a promising alternative to a paper resource handout as an initial, low-intensity strategy for providing tailored information about local food assistance programs. Over half of the reached cohort responded to text messages and one-third used the ZIP code–specific FindHelp community food resource link. This aligns with prior work showing that not all families with FI identified through screening desire assistance [45-47] and that patients are more likely to respond to text messages than click on embedded links [48]. Despite no preceding in-hospital interaction about FI resources, our text message response rate was similar to a pragmatic, automated, postdischarge SMS text messaging program that first contacted participants during admission, in which 55% of included patients responded to at least the first text message [49]. Applying caregiver interview data to the TAM-RLS framework, we hypothesize that this preference for text message use is due to (1) a need to engage through mechanisms that require only basic cellular service; and (2) greater trust in the confidentiality of responding to a text message as compared to clicking on an unfamiliar link. These findings suggest that future effective interventions that similarly provide tailored resources links to patients are likely to benefit from avoiding dependence on cellular data or internet and allowing two-way messaging interactions between patients and their care teams.

Though this study was not designed or powered to assess the effectiveness of the Text Connect intervention (eg, connection to a new food resource or subsequent health outcomes), text message respondents and interview participants reported getting a new food resource due to the information in the text message, suggesting that SMS text messaging can help a proportion of caregivers access food resources. By providing additional points of contact with families postdischarge that convey continued investment in their family’s well-being, the Text Connect intervention may also positively impact family and population health outcomes by fostering deeper trust in and connection between health systems and families they serve [50]. Caregiver interviews suggest that augmenting Text Connect by providing an option for caregivers to receive additional assistance through a follow-up phone call with a social worker or resource navigator may increase families’ food resource connections. This individualized assistance may also encourage connection to unique programs that exist in a caregiver’s community, such as Food is Medicine initiatives or other community partnerships. Improving access to nutritious foods for all children is critical to improving physical, mental, and developmental health outcomes. Thus, additional studies should rigorously compare the effectiveness of different methods of responding to positive FI screens, particularly attending to the effectiveness of different approaches across patient sub-populations.

### Limitations

Our findings should be interpreted in light of several key limitations. First, although automated EHR data collection and intervention related to food insecurity is feasible, low cost, and scalable, systematic monitoring is required to ensure system components are functioning as intended. We identified several Text Connect system issues in real time that resulted in occasional delays, pauses, and required manual import of records. However, the end user was unaware of these technical issues, so we expect minimal impact on their perception of acceptability or usability. The use of pragmatic data collection measures among the full cohort via Twilio was also a strength in that it lowered risk of response bias and facilitated more complete data collection. Second, though we saw a 50% text message response rate, we found a notable drop-off in completed responses as participants progressed through text message questions, limiting sample size and ability to draw conclusions about intervention effectiveness in facilitating connection to food resources. Additionally, though the screener used has shown 97% sensitivity for identifying families with FI [51], we likely missed a small fraction of caregivers with FI.

Finally, this study was conducted among patients on one unit and one service at a tertiary care, free-standing children’s hospital, and our results may not generalize to all caregivers with FI or other health systems. Future studies should test the feasibility of this intervention in other settings.

### Future Directions

Based on results of this study, the Text Connect intervention was approved by our health system as a method for providing food resource information to any family with FI identified during admission to the Children’s Hospital. We adapted message content in response to input from caregivers and are currently testing Text Connect’s effectiveness in a pragmatic randomized controlled trial (ClinicalTrials.gov; NCT06919445). Data from the next trial will inform cocreation of a stepped-care approach to resource connections that incorporates resource navigator follow-up for caregivers who are not engaged or assisted via text message alone.

### Conclusions

An automated text message is a feasible, usable, and acceptable way to provide families with FI identified during hospital admission with relevant food resource information. The Text Connect intervention is a system-level approach that was built with input from community members and can engage caregivers with varying levels of technology access. This approach is widely generalizable and could be adapted and implemented in other health systems that use modern EHR systems and have the resources to generate automated text messages to provide tailored content for FI and other health-related social needs (eg, housing insecurity).

## Supplementary material

10.2196/65852 Multimedia Appendix 1 Text survey content.

10.2196/65852 Multimedia Appendix 2 NO MORE FI interview script.

## Abbreviations

EHR: electronic health record
FI: food insecurity
mHealth: mobile health
REDCap: Research Electronic Data Capture
SNAP: Supplemental Nutrition Assistance Program
SUS: Simplified System Usability Scale
TAM-RLS: Technology Acceptance Model for Resource-Limited Settings
