# Supplementary material for: Development and Feasibility of a Novel mHealth Resource for Food Insecurity: Mixed Methods Cohort Study
Source: J Med Internet Res. 2025 Aug 26;27:e65852. doi: 10.2196/65852 (PMC12380405; doi:10.2196/65852)
Supplement: Multimedia Appendix 1 [file jmir-v27-e65852-s001.docx]

| **Text Content** | **Response** |
| --- | --- |
| One Day Follow Up  T1: Hello! VUMC recently sent you a text message with information on food resources. We are following up to understand how families are able to use this information. You may choose not to respond at any time.  Did you read the text message when you received it? Reply Yes or No  T2a: If ‘No’ **->**  Did you receive the text message with food resources? Reply Yes or No  T2b: If ‘Yes’->  With all you have going on in your life, how confident do you feel in being able to use this information to find food resources? (Reply with number from 1-5; 1= not confident to 5= very confident)   - With all you have going on in your life, do you intend to use this information to find food resources? (1= definitely do not intend to use to 5= definitely intend to use)   T3: Any response 🡪 Thank you! We will follow up again in 2 weeks. | Yes/No  Yes/No  1-5  1-5 |
| Two Week Follow Up  T1: Hello! We are following up about the text message with food resource information you recently received from VUMC. You may choose not to respond at any time.  Have you used this information to find food resources? Reply Yes or No  T2a: If ‘No’ **->**  Did you receive the text message with food resources? Reply Yes or No  T2b: If Yes ->   - Did you use a resource (i.e. pick up food at food pantry, enroll in SNAP or WIC)? Reply Yes or No     T3: If ‘Yes’ ->   - How easy was it to use this information to find resources? Reply with number from 1-5; 1=very easy to 5=very hard | Yes/No  Yes/No  Yes/No  1-5 |
