# Supplementary material for: Development and Feasibility of a Novel mHealth Resource for Food Insecurity: Mixed Methods Cohort Study
Source: J Med Internet Res. 2025 Aug 26;27:e65852. doi: 10.2196/65852 (PMC12380405; doi:10.2196/65852)
Supplement: Multimedia Appendix 2 [file jmir-v27-e65852-s002.docx]

Thank you for agreeing to participate in this survey. During this interview, I would like to ask you about your experience with a text message with information on food resources you received after discharge from Vanderbilt Children’s Hospital. We are trying a new way to get information about food resources to families. The text messages are designed to deliver resource information in a private way that allows families to search directly for food resources near where they live.

There are no right or wrong answers, so please be as honest as possible in telling us about your experience, including what you liked and what could be better.

Is it ok if I start the recorder now?

First, based on your experience receiving the text message with food resource information, I want to hear what you think about using text messages to provide resource information to families.

1. What might families like about getting food resource information through a text message?
2. What might families not like about getting food resource information through a text message?

Next, I want to talk about how receiving resource information through a text message compares with some other methods of getting resource information.

1. We are currently also providing a paper handout with food resource information at the time of leaving the hospital. What might families think about getting food resource information through a text message compared to a paper handout? (*Probes:* *What are benefits, disadvantages, challenges of each? Is one better than the other- why?)*
2. Some hospitals also have individuals who are familiar with resources in the community, called community navigators, who can meet with families to help connect them to food resources. What might families think about getting food resource information by meeting with a person compared to getting information in a text message? (*Probes: What are benefits, disadvantages, challenges of each? Is one better than the other- why ? Any benefit to receiving information anonymously without needing to interact with providers/staff?)*
3. What method, or combination of methods, (such as a paper handout, text message, meeting with a person) would be most helpful for families? *Probe: What do you think is most helpful about this approach?*
4. Is there another way to connect families to food resource information that we have not discussed?
5. How would families feel about Vanderbilt sending their child’s primary doctor a notification that they received food resource information from the hospital? *(Probe: Would this be helpful- why? Any concerns about sharing this information?)*

Now I want to talk specifically about the text message with food resource information that you received.

1. Do you remember receiving a text message with information on food resources?
   1. Yes
   2. No

(For interviewer, complete question from NO MORE FI project)

Did patient respond to any of the 1 day or 2 week follow up text messages?

Yes

No

We also sent a text message one day and two weeks after you received the food resource information asking whether you had read the text message or used it to look for resources. The purpose of these messages was to get input from families on whether the text message information was helpful.

1. Did you receive those text messages?
   1. Yes
      1. Can you tell me more about why you didn’t respond to them? *(Examples if needed: read it and forgot, didn’t know what the messages were about, etc)*
      2. Is there anything we could change about the follow up messages to make you more likely to respond? (For example, the time of day they are sent, how long after the initial text message are sent, content of the message/explanation for why they are being sent)
   2. No
      1. If you are able to look back in your phone using the number 615-880-9815, do you see any messages that you might have missed initially? *(For reference if needed: The first one-day follow up message reads:* Did you read the text message with food resources when you received it? Reply Yes or No. *The first 2-week follow up message reads:* Have you used this information to find food resources? Reply Yes or No)
         1. Yes
         2. No
      2. Thank you for checking! That helps us know if there are issues with our messages getting delivered to patients.

Would it be helpful for me to read the content of the text message as a reminder of what was included? You should also be able to search your phone for the message using the number 1-615-880-9815 to look back at the content.

*If participant requests to hear a reminder of the text message content, interviewer reads from ‘Text Connect Message Document’.*

I'm going to read you several statements that describe the text message with food resource information that you received. On a scale from 1 to 5, where 1 is Strongly Disagree and 5 is Strongly Agree, please tell me how much you agree with each statement. Let me know if you need a reminder of the answer choices at any time.

1. I would use the information in the text message to look for food resources again.
2. The text message is too complex for me.
3. The text message was easy to use.
4. I really need help from someone to use the information included in the text message.
5. The various types of information in the text message were combined well.
6. The information included in the text message was confusing for me.
7. Learning to use the text message to look for resources was quick for me
8. The text message information was hard to use.
9. I felt confident using the text message to look for food resources.
10. The text message is an acceptable way to get food resource information.

After reading all items, go back and request further information on any item with a flagged response indicating problems

Can you please tell me why you chose 'Disagree' for the following statement?

Can you please tell me why you chose 'Agree' for the following statement?

Now, I'll ask you some questions about your experience receiving and using the text messages with food resources that you received from Vanderbilt.

First, we’ll talk about your experience receiving the text messages and the content included in the messages.

1. How soon after receiving the initial food resource text message did you read it?
2. Same Day
3. Following Day
4. Not until after receiving the reminder food resource text message
5. Never
6. I don’t remember
7. Was anything about the message confusing? (i.e. information included in the text message, where it came from, why it was received, etc.)
   1. Yes
      1. Can you tell me about what was confusing? How could we change it to make it easier to understand?
   2. No
   3. I don’t remember
8. Did you have any technical issues with the text messages? For instance, did the text messages arrive out of order, nothing happened when you clicked on the included links, etc.?
   1. Yes
      1. Can you tell me about the issue(s) you experienced?
   2. No
   3. I don’t remember
9. Is there any information you would add to the text message?
10. Is there any information you would take out of the text message?
11. Currently, we are sending an initial food resource text message 1 or 2 days after leaving the hospital. Would you change the timing of when the text messages are sent?
    1. Yes
       1. When would be a better time to receive this information? Why?
    2. No

Finally, we’ll talk about using the information in the text messages you received from Vanderbilt to search for and connect with food resources.

1. Did you use any of the following information included in the text message to search for food resources? Did you … (Select all that apply)
   1. Text FEEDS to the number included to receive a list of food pantries near your ZIP Code
   2. Search for resources in your ZIP Code through the website link included
   3. Click on the website link for information on SNAP
   4. Call the Second Harvest Food Bank phone number for assistance completing a SNAP application
   5. None of the above

2a. If ‘Yes’ to any of the above:

1. Which information was most helpful?
2. Which information was least helpful?

2b. If ‘Yes’ to b (search for resources using link)

1. What did you think about using this site to look for resources near your home? *(Probe: Was the information included helpful- why? Was it easy to use?)*

2c. If ‘None of the above’

- 1. Can you tell me more about why you did not use any of the information?
  2. Were there any specific challenges to using the information? (Skip to #3) *(examples if needed: unfamiliar with the technology, no access to wifi, etc)*

1. Did you use any of the information included in the text message to get a food resource? By this I mean picking up food from a pantry, completing an application for SNAP, etc.
   1. Yes
      1. What kind of resource(s) did you get?
      2. Was this the first time you have *“picked up food from that location”; “completed a SNAP application”, etc tailored to resource mentioned.*
      3. How soon after receiving the text message did you get “*resource mentioned above”*?
      4. Did you face any challenges in the process of getting “*resource mentioned above”*?
         1. Yes
            1. What was challenging?
            2. Is there anything that would have made it easier to get “*resource mentioned above”*? *(Ie, childcare, transportation assistance, etc.)*
         2. No
   2. No
      1. What challenges did you face in using the information in the text message to get a food resource? *(Probe: For example, some families may have trouble getting transportation to a food pantry, etc)*
      2. Can you think of anything that would have helped you use the information in the text message to get a food resource?
2. Before we finish, is there anything we haven’t talked about that you would like to share?

Thank you completing this interview. We appreciate your participation and feedback to help us improve this program. While I have you on the phone, let me make sure I have the correct address of where we should mail your participation gift card.
